# Expanding preconception carrier screening for the Jewish population using high throughput microfluidics technology and next generation sequencing

**DOI:** 10.1186/s12920-016-0184-7

**Published:** 2016-05-13

**Authors:** Moran Gal, Khen Khermesh, Michal Barak, Min Lin, Hadas Lahat, Haike Reznik Wolf, Michael Lin, Elon Pras, Erez Y. Levanon

**Affiliations:** The Mina and Everard Goodman Faculty of Life Sciences, Bar-Ilan University, Ramat Gan, 52900 Israel; Fluidigm corporation, South San Francisco, California; The Danek Gertner Institute of Human Genetics, Sheba Medical Center, Tel Hashomer, Israel; Sackler Faculty of Medicine, Tel Aviv University, Tel Aviv, Israel

**Keywords:** Carrier screening, Next generation sequencing, Microfluidics, Genetic testing, Jewish population

## Abstract

**Background:**

Genetic screening to identify carriers of autosomal recessive diseases has become an integral part of routine prenatal care. In spite of the rapid growth of known mutations, most current screening programs include only a small subset of these mutations, and are performed using diverse molecular techniques, which are generally labor-intensive and time consuming. We examine the implementation of the combined high-throughput technologies of specific target amplification and next generation sequencing (NGS), for expanding the carrier screening program in the Israeli Jewish population as a test case.

**Methods:**

We compiled a panel of 370 germline mutations, causing 120 disorders, previously identified in affected Jewish individuals from different ethnicities. This mutation panel was simultaneously captured in 48 samples using a multiplex PCR-based microfluidics approach followed by NGS, thereby performing 17,760 individual assays in a single experiment.

**Results:**

The sensitivity (measured with depth of at least 50×) and specificity of the target capture was 98 and 95 % respectively, leaving minimal rate of inconclusive tests per sample tested. 97 % of the targeted mutations present in the samples were correctly identified and validated.

**Conclusion:**

Our methodology was shown to successfully combine multiplexing of target specific primers, samples indexing and NGS technology for population genetic screens. Moreover, it’s relatively ease of use and flexibility of updating the targets screened, makes it highly suitable for clinical implementation. This protocol was demonstrated in pre-conceptional screening for pan-Jewish individuals, but can be applied to any other population or different sets of mutations.

**Electronic supplementary material:**

The online version of this article (doi:10.1186/s12920-016-0184-7) contains supplementary material, which is available to authorized users.

## Background

Genetic screening to identify carriers of recessive diseases (also known as preconception, premarital, prenatal, or reproductive screening) aims to detect couples at risk for transmitting hereditary genetic diseases to their offspring. The purpose of such screening is to provide the prospective parents the opportunity to make informed decisions regarding their reproductive options or the use of early interventions (when available). From a public health perspective, carrier screening programs reduce the incidence of genetic diseases, and are cost-effective from medical expenditure aspect.

The introduction of NGS platforms in recent years enables DNA sequencing at an unprecedented yield and low cost. It is clear that this technology will become the method-of–choice for any clinical genetic testing program and it holds the potential of expanding the current screening programs for reproductive choices, in a similar manner that mass spectrometry has enabled screening of newborns for metabolites [1]. Despite the feasibility of sequencing entire exomes and genomes, such an approach is still not affordable or applicable for population screening, in which thousands of samples must be analyzed and unambiguous results must be obtained automatically in a timely manner. Thus, for the foreseeable future, a targeted re-sequencing method is still required. Moreover, the screening techniques chosen should be flexible and scalable, in order to keep up with the new genetic discoveries and rapid growth of mutation databases.

**Fig. 1:**
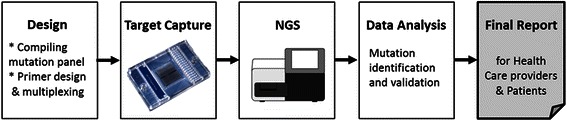
The general workflow of this study which contains four principal steps: I. Data acquisition and design - creating the panel of mutations to be targeted and designing specific primers and their multiplexing, II. Experimental procedures for target capture (using Fluidigm microfluidics device) and sequencing (MiSeq platform), III. Bioinformatics analysis, mutation identification and validation and IV. Providing final report with the mutations identified in each sample

In this work we explored the feasibility of microfluidics technology for target capture, followed by NGS to expand carrier screening for reproductive purposes (Fig. 1). The Jewish population in Israel was chosen as a test case. Carrier screening in the Jewish population for reproductive purposes has been carried out for decades. Since the first preconception carrier screening program for Tay Sachs disease was introduced for Ashkenazi Jews in the 1970s [2], additional common mutations for other severely debilitating diseases in the Jewish population were identified, and have been introduced into screening panels [3–5]. As a result, to date, the birth of affected children with these diseases is rare, and the screening programs have essentially become the “standard of care”, administered either premarital, before conception, or during the pregnancy.

An updated catalog of disease-causing mutations found in Jewish population contains more than 1000 different mutations responsible for hundreds disorders (http://server.goldenhelix.org/israeli/) [6]. Yet, currently in most centers in Israel, genetic testing is offered for only a limited subset of these (~30 diseases). The major limitations for introducing new mutations into screening panels are financial and technological constraints, such as current laboratory techniques, human labor and test execution time.

The official recommendations for genetic screening in Israel from the Israeli Society of Medical Genetics [7] and the Israeli Ministry of Health (MOH) [8] (and its public funding) are largely based on the frequencies of the mutations in the population (i.e. testing is prioritized for more common mutations and avoided for the rarer ones), and the screening is performed in an ethnic-dependent manner (i.e. different mutation panels for Ashkenazi Jews, Jews originating from North Africa, Jews from Iraq, etc.).

However, over the past decades, the inter-ethnic marriage rate has increased among the Jewish population in Israel [9], and thus, choosing the relevant set of genetic disorders for each couple from a growing list of available tests, is becoming an impractical task.

The transition to high throughput technology-based screening will most probably obviate both the mutations’ frequency criteria and the ethnicity-dependent recommendations applied today, as was also acknowledged in the new ACMG policy [10]. In this study, we provide proof-of-concept for combining high-throughput technologies for targeting and sequencing a set of hundreds known mutations, tailored to a specific population, to be use in pre-conception carrier screening.

## Methods

### Target selection and compiling a mutation panel set

We compiled an updated comprehensive list of disease-causing mutations in the Jewish population from data provided by the Department of Community Genetics at the Israeli Ministry of Health (as of Oct 2011). In the targeted panel we included mutations for Autosomal recessive (AR) or X –linked disorders, in which genetic testing can identify couples at risk, and is likely to influence parental decisions regarding the pregnancy or the clinical management of the newborn.

The online resources used for clinical evaluation included the booklet “Mendelian disorders among Jews” from the MOH website [8], “OMIM” [11] and “GeneReviews” at the NCBI website [12] Human Gene Mutation Database (HGMD) [13], and relevant literature.

More than half of the mutations in the panel were described in non-Ashkenazi patients from various countries of origin. This set of mutations better reflects the current Israeli population, where Jews of non-Ashkenazi origin comprise more than half of the Jewish population (personal communication from the Israeli MOH).

While some of the listed mutations have clear support for being founder mutations (i.e. were identified in several unrelated families), a large proportion were found only in single families, and are currently regarded as “private” mutations. However, we could not exclude the possibility that they do exist (with lower frequencies) in other individuals, and therefore we included them in the panel. The final panel comprised of 370 mutations of both single nucleotide variants (SNVs) and small insertions and deletions (indels) in 148 genes associated with 120 disorders (Additional file 1).

Two of the mutations were substitutions of the same genomic nucleotide to different alternative alleles (both resulting in pathogenic mutations): p. R261P and p.R261Q in the *PAH* gene and p.P339H and c.1016insC in the *CYBB* gene. For the analysis of coverage depth, each of the pairs were counted as one thus in total we had to target 368 genomic positions for 370 mutations.

We also included in the panel four mutations of large-rearrangements (deletion or insertion of up to tens of Kb), with characterized genomic breakpoints, but these were eventually excluded from the final analysis. The genomic positions of all mutations were verified, including the precise position of breakpoints in gross insertions and deletions.

### Primer design and multiplexing

To capture the genomic positions of the mutation list, 345 primer-pairs were designed and multiplexed into 45 sub-groups containing 1-12 primer pairs in each pool using Fluidigm custom primer design service. To test the ability to capture and sequence large rearrangements, four primers-pairs were designed to span the breakpoints of four mutations of large deletions or insertions. These primers were expected to capture the abnormal chimeric products that exist only in carriers’ genomes (See examples in Additional file 2).

The primers multiplex algorithm (Fluidigm Co.) evaluated the primer-primer interactions in order to minimize the generation of undesirable PCR products, but to maximize PCR specificity and efficiency. The major considerations were to avoid combining primer pairs that produce PCR products that overlap or are separated by less than 5 kb, have high variability of GC content or are not within 20 % of the average size. Amplicon lengths were between 64 and 200 bps, with one outlier amplicon of 418 bp designed for a large deletion mutation (6.7 kb deletion in PAH gene). 54 mutations were designed to be capture by multiple primer pairs, and 49 amplicons covered multiple mutations.

### DNA samples

The study was approved by the ethics committee of Sheba Medical Center, Israel.

We obtained genomic DNA samples from 43 healthy anonymous carriers of various genetic disorders. These carriers were previously identified by traditional genetic assays, and were used as positive controls to examine the method. We included eight biological replicates (different samples that carry the same mutation) and four technical replicates (the same sample tagged with two different barcodes). The control samples were chosen to include all types of genetic variations: 34 single nucleotide substitutions (SNVs), 16 small indels, and five gross re-arrangements, all analyzed in a blinded manner (Table 1, “Knowns”).

**Table 1:** Summary of all mutations identified in the experiment

“Knowns”			
Sample	Gene	Mutation	Detected
S3	HEXA	c.1278insTATC	√
S6	CFTR	p.F508 del	√
S7	DYSF	c.1624delG	√
S8	FANCA	c.2172-2173 + G	√
S9	GBA	p.84dupG	√
S10	GJB2	c.167DelT	√
S10	CFTR	p.N1303K	√
S11	GJB2	c.35delG	√
S11	PAH	A403V	√
S11	ATM	p.103C > T	√
S12	GJB2	51_62del12ins1	√
S13	HEXA	c.1278insTATC	√
S13	SMARCAL1	IVS4 -2 A > G	√
S14	HEXA	p.F304/305del	√
S15	SPMD1	p.R610del	√
S15	PEX6	p.A809V	√
S16	BCKDHB	p.R183P	√
S17	CERKL	IVS1 + 1G > A	√
S18	CFTR	c.405 + 1G > A	√
S19	CFTR	p.G542X	√
S20	CFTR	p.G85E	√
S21	CFTR	p.W1282X	√
S22	FANCC	IVS4 + 4A > T	√
S23	G6PC	p.R83C	√
S24	GBA	c.115 + 1G > A	x^a^
S25	GBA	p.N370S	√
S25	GBA	p.V394L	√
S25	PEX6	p.A809V	√
S26	GBA	p.R496H	x
S28	CFTR	p.Q359K + p.T360K	√
S30	IKBKAP	c.2204 + 6 T > C	√
S31	HEXA	p.G269S	√
S32	TMEM216	p.R73L	√
S33	HEXA	IVS12 + 1G > C	√
S34	HEXA	p.R170Q	√
S35	SPMD1	p.L302P	√
S38	SPMD1	p.R496L	√
S39	HEXA	p.G250V	√
S40	ATM	p.103C > T	√
S41	CFTR	p.N1303K	√
S42	GJB2	c.167DelT	√
S42	CFTR	p.N1303K	√
S43	HEXA	c.1278insTATC	√
S44	DYSF	c.1624delG	√
S45	GJB2	51_62del12ins1	√
S46	CFTR	p.N1303K	√
S47	GJB2	c.35delG	√
S47	PAH	p.A403V	√
S47	ATM	p.103C > T	√
S48	CFTR	p.Y1092X	√
Large Rearrangements			
S1	GALT	Del 5Kb	√^b^
S4	PAH	Del 6.7Kb	√^b^
S3	MAK	Ins353bp	√^b^
S29	MAK	Ins353bp	√^b^
S32	MAK	Ins353bp	√^b^
“Unknowns”			
Sample	Gene	Mutation	validated
S3	CLRN1	p.N48K	√
S3	ASPA	p.E285A	√
S7	AMN	c.208-2A > G	√
S17	SAMD9	p.R344X	√
S18	GUCY2D	c.389delC	NA
S18	SERPINA1	p.E342K	√
S18	SERPINA1	p.E264V	√
S22	GJB2	p.V37I	√
S31	ABCC8	c.3989-9G > A	√
S31	PEX6	p.A809V	√
S33	SPMD1	p.R496L	√
S39	FANCC	IVS4 + 4A > T	√
S40	CFTR	p.L997F	NA
S40	FAM161A	c.1355_6delCA	√
S44	EYS	p. Thr135LeuX25	√
Variants with high incident			
Gene	mutation	samples	
TRMU	p.A10S	S10,S11,S16,S33,S38,S42,S44,S47	
ACADS	p.G185S	S2,S4^c^,S8,S12^c^,S13,S51,S18^c^,S19,S21,S22,S23^c^,S24,S26,S28,S33,S35,S36^c^,S39,S41,S44,S45^c^,S46,S48	
MYOC	p.R76K	S11,S12,S13,S16,S18,S24,S28,S29,S31,S45,S47	
LIPA	p.G5R	S2,S5^c^,S6^c^,S29,S37,S44,S48	

^a^Ideantified manually with 26 % mutant allele

^b^detected by finding exact match of chimeras sequences (explained in the Methods)

^c^found as homozygous

### Target capturing, library preparation and high-throughput sequencing

Genomic DNA templates were amplified using the 48.48 Access Array IFC, according to the manufacturer’s instructions (Fluidigm). Briefly, 48 DNA samples were combined with the 48 multiplex primers pairs on a microfluidic IFC-chip. The IFC-chip was loaded with PCR reagents using the Access Array pre-PCR controller loading script and upon completion it was transferred to a thermocycler. After amplification, pooled amplicons from each of the 48 DNA templates were harvested using Access Array post-PCR controller harvesting script, and used as input for the subsequent off-chip PCR reactions, in which each mini-library underwent tagging with sample specific barcodes and attachment of sequencing platform-specific adapters. The output libraries were purified using Qiagen PCR purification kit and analyzed on an Agilent DNA1000 BioAnalyzer chip. The purified combined libraries were sequenced using MiSeq 150 bps pair-end protocol, according to the manufacturers’ standard instructions.

### Bioinformatics analysis

MiSeq sequencing data was de-barcoded to samples by the Illumina BaseSpace server.

Alignments to the human genome (hg19) were performed with BWA version 0.7.4^16^, applying the mem argument. Depth calculations per target and sample were done with the DepthOfCoverage tool of the GATK toolkit [14].

Three samples were excluded from further analysis of variant calling and depth of coverage statistics: sample 2 and sample 27 had the lowest reads counts (35,142 and 118 reads, less than 1 % of reads in each of the other samples), pointing to poor DNA quality or technical pipetting problem during library preparation; sample 36 was excluded due to insufficient coverage of large number of targets (44 targets with less than 10X), possibly due to technical microfluidics malfunction in specific wells of the Access Array.

Single nucleotide variants (SNVs) and small indels were identified with the GATK toolkit version 3.1.1 with default parameters (including realignment and recalibration steps) [14]. Variants detected in technical replicates were mostly identical, and almost all discordant ones were in poorly covered positions (DP < 10) in one of the duplicates. Variant Call Format (VCF) files were then intersected with a list of positions of the mutations of interest. For small insertions or deletions, a search within adjacent genomic intervals was performed, and positions were manually curated as needed.

Variants detection criteria were minimal depth of coverage (DP) of at least 50X and mutant allele percentage between 30 and 70 % for calling heterozygotes.

Mutations of large re-arrangements were detected by identifying reads that align to mutant (chimeric) junction reference sequence (could be captured only in samples of carriers, see Additional file 2).

### Validation of mutations

Novel and unexpected mutations that were identified in the experiments (i.e. not previously known to be present in the samples) were validated by Sanger sequencing in ABI PRISM 3730xl Genetic Analyzer (Applied Biosystems, Warrington, UK).

## Results

### Target Enrichment Performance

Forty-eight DNA samples were captured for 368 genomic positions (of 370 mutations) using the combined high-throughput methods of microfluidics based multiplex PCR and NGS.

Sequencing the captured library with Illumina’s MiSeq yielded a total of 5.4Gb with quality score of ≥ Q30 (i.e. sequencing error probability of 1 in 1000) in 92.1 % of bases. 33,241,984 reads could be assigned to specific samples by their barcodes. The average reads count was 692,541 per sample (range 392,815-1,455,638, excluding two samples, see methods for details).

Average alignment rate to the human genome was 99.8 % per sample (range 96–100 %), with 95 % of reads mapping to the expected amplicons, indicating high specificity of the primer design and capture procedure. 365 of 368 (99 %) genomic positions of the targeted mutations were covered by at least 1 read, indicating successful target capture by nearly all primer-pairs.

**Fig. 2:**
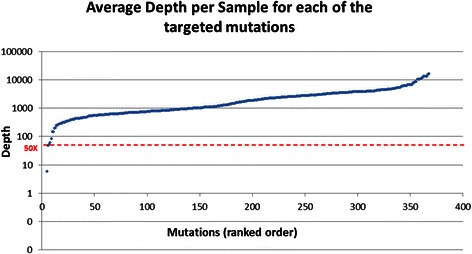
Average Depth per sample for each targeted mutation in ranked order (logarithmic scale). 353 out of 368 (99 %) mutation positions were covered by an average at least 50X in a sample. Four mutations with average of zero coverage are not presented in the graph

As the purpose of the experiment is to detect heterozygote carriers of these mutations, sufficient coverage at the mutation genomic position must be achieved. After excluding three samples due to technical problems (see Methods), the sensitivity of the capture protocol was very high, as 358 of 368 (97 %) of the targeted genomic positions had an average depth per sample of at least 150X, with more than 50X in all samples (Fig. 2).

**Fig. 3:**
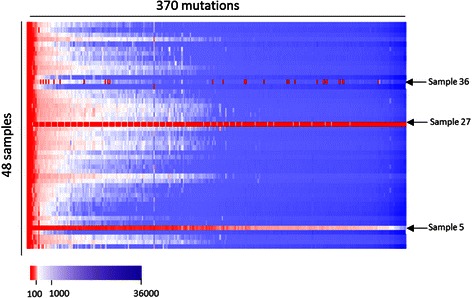
Heat map showing the depth of coverage of each mutation in each of the samples. A matrix of 17760 assays (368 mutation positions X 48 samples) is presented with color coded of red for less than 100X (our defined threshold) and white to purple gradient for more than 100X from low to high coverage, respectively. The mutations are presented in ranked order by their average sample depth. Marked with arrows are the three samples that were excluded from analysis due to technical problems (see Methods)

The target capture with the multiplexed microfluidics PCR technology enabled to essentially perform 16,650 independent assays in this experiment (where “assay” defined as determining the presence of a single mutation in a specific sample)., Reassuringly, the average depth of an assay in our experiment was 2511X, with 98 % of the assays covered by at least 50 - reads. The depth of coverage of most targets was uniform across the samples (Fig. 3).

From the tested individuals (single samples) aspect, an effective and well-designed high-throughput carrier test should have minimal inconclusive results, i.e. number of mutations that cannot be confidently identified due to low coverage (under a pre-defined threshold). Excluding the technically failed samples (see Methods), the minimal percentage of conclusive tests, with DP threshold of at least 50X in a sample, was 98 % (360/368) showing high completeness also per each sample analyzed.

Of note, most of the low covered targets were consistent across samples, indicating that better primer design might increase their capture efficiency.

### Variant detection

To assess the ultimate goal of this protocol -its capability to detect the mutations of interest- we used 40 samples of known heterozygote of 50 different mutations (both SNVs and indels). These samples were previously identified by traditional methods routinely applied for carrier screening.

The samples were blindly screened using GATK variant calling pipeline, and 48 (96 %) of these mutations were successfully called in their expected samples and in heterozygotes state (Table 1, “Knowns”). The two false negatives were mutations in the GBA gene (R496H and c.115 + 1G > A), causing Gaucher disease. Identifying mutations in this gene has been recognized as problematic due to the presence of the paralogues pseudogene GBAP1 [15, 16]. The high similarity between GBA and GBAP1 pose difficulties in specific capture and accurate mapping of reads, and challenges mutation detection, especially where GBAP1 variants can be confused with GBA mutations [17]. For example, in the c.115 + 1G > A mutation, the mutated allele (A) corresponds to the actual normal reference sequence of the pseudogene, so that reads originated from the GBAP1 can be identical to ones derived from carrier with mutated GBA. Manual inspection for this mutation had revealed that the mutant allele is present with allele frequency of 26 % at the sample of a known carrier, as expected if the reads from GBA and GBAP1 were captured in even ratio.

Nevertheless, we correctly identified other mutations in this gene (e.g.: p.84dupG in sample 9, p.N370S and V394L in sample 25) captured by other amplicons, proving that with careful primer design, adjusting read length and ascertainment of phasing during the bioinformatics analysis, mutations in such genes can be identified.

In addition to the expected mutations described above, we detected 64 additional variants from the target list that were not previously identified in the samples (Table 1,”Unknowns”). 49 of these were accounted for by four substitutions, each identified in multiple samples: p.G5R in LIPA.p.A10S in TRMU, p.G5R in LIPA p. R76K in MYOC and p.G185S in ACADS which were identified in 7,8,11 and 23 samples respectively; in some samples they were called as homozygous (Table 1,”Unknowns - variants with high incident”). These observations support their re-classification as common and benign polymorphisms, and we suggest that they should not be included in future versions of the mutation panel. The other 15 unexpected mutations were found each in a single sample; 13 out of 13 were validated by Sanger sequencing or other methods.

In total, we were able to validate 61/63 (97 %) of the mutations that were identified in the experiment. The Average DP in these mutations was 519X and the average percentage of mutant allele was 49 % (range 36–61 %, Additional file 3).

Finally, we also tested the ability of the capture protocol to identify mutations of large re-arrangements with known breakpoints, using five samples of carriers and special designed primers. We successfully identify reads that aligned to mutant junction reference sequences in those samples, proving that such mutations type could also be included in the panel (see examples in Additional file 2). However, as these mutations were called with insufficient depth, it requires further calibration.

## Discussion

We explored the feasibility of coupling microfluidics technology for target capture with NGS as a fast and cost-effective method for performing prenatal carrier screening of the Israeli Jewish population. Our protocol enabled performing ~17,000 assays in single experiment, in each we aimed to determine the presence of a specific mutation in a specific sample. The results of the pilot study presented here were promising in both aspects of target capture performance and the accuracy of mutation detection.

The capture efficiency was very high as 97 % all of the targeted mutation’ positions were captured with at least 50X in all samples (excluding the three faulty samples). The sensitivity, measured as the percentage of mutations that were covered in adequate depth (50X) to enable reliable variant identification, was 98 % both per individual sample tested and globally, i.e. the total assays produced on the chip. This minimizes the need to repeat samples or perform supplemental analysis by other methods such as Sanger sequencing. Finally and most importantly, we were able to detect 96 % all mutations present in the positive control set, and validate all the newly discovered ones in the validation set.

One of the major advantages of the microfluidics device is the lack of potential cross hybridization, which was reflected in the very low proportion of off-target reads (high specificity). Another advantage of this target capture technology is its dynamism and flexibility. Unlike fixed genotyping methods, such as array-based hybridization probes [18], the panel set can be easily updated by changing the composition of primers in the microfluidics device. New mutations are constantly been discovered, and the panel can be adapted to keep pace with these findings.

Undoubtedly, NGS is becoming the method of choice for performing genetic testing in a clinical setting. Other studies utilizing NGS for carrier screening for AR diseases have been published. Bell et al. captured and sequenced a subset of the exome (containing only genes that were previously associated with AR diseases) [19] and Umbarger et al. focused on sequencing the entire coding region of 15 genes [20]. In contrast, we intentionally focused on a specific set of mutations. In our view, although the cost of sequencing entire exomes or genomes is constantly dropping, there are several advantages in using a targeted approach.

First, in large population screening, such as prenatal carrier screening in Israel, tens of thousands of samples are tested per year, and results must be obtained automatically and in a timely fashion. For reliable variant calling that meets clinical standards, sequencing data of both high quality and depth is required. Achieving these parameters in large genomic regions (such as exomes or genomes) and in large population samples is not yet cost-effective. Apart from the sequencing itself, there are also costs involving the computational burden of analyzing and storing large data sets, providing additional advantage to approaches that generate a relatively low and manageable amount of data.

Second, the fine-targeted approach is highly suitable for screening genetically homogenous or isolated populations, such as the Jewish individuals in Israel, in which known mutations in specific genes account for the majority of the carriers [21, 22].

Third and most fundamental, is the concern regarding ambiguous results. In any method that is not constrained to identifying already known mutations, the possibility of identifying variants with uncertain significance (VUS) is inevitable. Determining the biological and clinical significance of such variants is in many instances difficult and may yield inconclusive results. Not every presumed loss-of-function variant has disease-causing potential even in well annotated Mendelian genes, such as CFTR [23] or HEXA [24]. Moreover, elucidating VUS requires a personalized approach to data interpretation, which is beyond the primary purpose of large population screening, and may impose major difficulties for healthcare providers that have to convey the genetic information to the prospective parents. While broader-scaled sequencing is advantageous in a research setting, or in clinical testing of affected individuals, it still lacks sufficient clinical validity and utility for screening healthy individuals. In our view, clinical screening should identify only variants that are unequivocally disease associated and whose detection in carrier screening is expected to aid in reproductive decisions or lead to early intervention. In their recent policy statement on expanding prenatal/preconception carrier screening with high-throughput technologies, the American College of Medical Genetics has raised these concerns and stated that the tests should “include specific citations that support inclusion of the mutations for which screening is being performed” [10].

For these reasons, we suggest that for the purpose of pre-conception screening, a fine targeted method is necessary, and have included in our panel only mutations that were previously reported to be disease causing.

In this mini-cohort of 48 samples, we have identified four variants that are unlikely to be associated with any pathology. When compiling the mutation panel set, these variants were included because previous publications described them as disease-associated mutations. Two variants (p.A10S in TRMU and.G185S in ACADS) also appear in HGMD [13], and are flagged as “clinically associated” in dbSNP, p.R76K in MYOC was believed to contribute to glaucoma in a di-genic inheritance [25] and p.G5R in LIPA was shown in a functional assay to cause reduced enzymatic activity [26]. These variants were highly prevalent in our sample set, were identified as homozygous in some of the samples, and are also present in 1000 Genomes with minor allele frequency of at least 10 %. Therefore, we suggest that these variants should be re-evaluated and considered as benign polymorphisms, and be excluded from future panels. Similar findings in other reported mutations were also observed by Bell et al [19], highlighting the need to improve databases of clinically associated variants. Nevertheless, these findings illustrate the strength of our screening protocol. By simultaneously screening dozens of samples for each of the mutations, rapid information about the frequencies of all tested mutations will accumulate, and this in turn can help to correct such misinterpretations and to optimize screening recommendations.

Before implementing our protocol into clinical care, a few improvements are required. First, mutations and disorders included should be updated to be compatible with the latest professional guidelines and databases. Second, for the poorly covered mutations and the large rearrangements primers should be re-designed. Third, redundant amplicons for each mutation can be introduced to minimize technical capture failures. Fourth, the bioinformatics analysis for mutation detection should be fine-tuned, especially in challenging genomic regions such as genes which have pseudogenes. In addition, In light of the excessive depth achieved in our experiment for some of the mutations, further multiplexing of primers for additional mutations and/or increasing the number of samples in the parallel sequencing can be considered, to further reduce the cost per sample and per mutation.

## Conclusions

This is a proof-of-concept study designed to perform large pre-conception population screening for thousands of genetic tests in single experiment, without compromising on high confident of variant detection.

Our experimental system is practical and cost-effective, balancing the high-throughput potential of the latest cutting-edge technologies and the evidence-based knowledge that is crucial in a clinical setting. The current version of the protocol can be implemented into a clinical service after few modifications. Moreover, this approach can be also be customized for screening any other populations with different mutation profile.

## Additional files

Additional file 1: List of mutations included in the screening panel. (XLS 61 kb) Additional file 2: Examples of primer design and supporting reads for the large-rearranged mutations. A. Deletion of ~5Kb in the GALT gene, leading to Galactosemia. This mutation is composed of four breakpoints, leading to two large deletions and one small insertion, and resulting in the loss of almost the entire gene (adapted from Coffee et al. [27]). Vertical arrows depict the breakpoints, and horizontal arrows mark the primers used for capture. Primers 1 F +1R are used to capture the amplicon created in the 5′ deleted region, and the 2 F + 2R primers are used to capture the amplicon created in the 3′ indel region. B. Insertion of a 353 bp Alu element into the MAK gene leads to Retinitis Pigmentosa (found by Tucker et al. [28]). (PNG 69 kb) Additional file 3: Total depth and percentage of mutant reads in the validated heterozygote mutations (both previously knowns and unknowns). As expected, the percentage of mutant alleles approximated 50 % for both SNPs and indels. (PNG 17 kb)
