# Supplementary figures and images for: Expanding preconception carrier screening for the Jewish population using high throughput microfluidics technology and next generation sequencing
Source: BMC Med Genomics. 2016 May 13;9:24. doi: 10.1186/s12920-016-0184-7 (PMC4865987; doi:10.1186/s12920-016-0184-7)

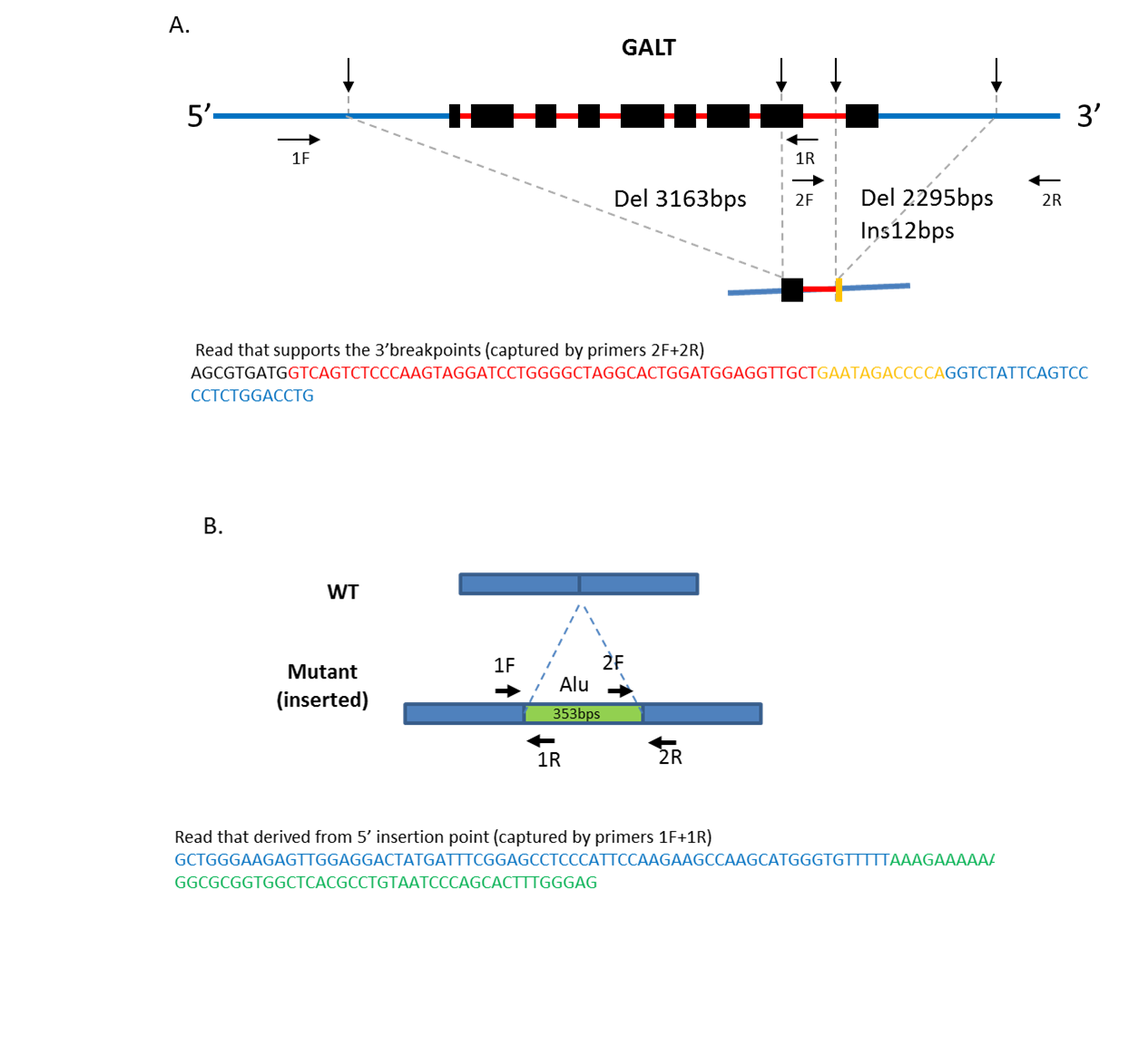

Supplement: Additional file 2: — Examples of primer design and supporting reads for the large-rearranged mutations. A. Deletion of ~5Kb in the GALT gene, leading to Galactosemia. This mutation is composed of four breakpoints, leading to two large deletions and one small insertion, and resulting in the loss of almost the entire gene (adapted from Coffee et al. [27]). Vertical arrows depict the breakpoints, and horizontal arrows mark the primers used for capture. Primers 1 F +1R are used to capture the amplicon created in the 5′ deleted region, and the 2 F + 2R primers are used to capture the amplicon created in the 3′ indel region. B. Insertion of a 353 bp Alu element into the MAK gene leads to Retinitis Pigmentosa (found by Tucker et al. [28]). (PNG 69 kb) [file 12920_2016_184_MOESM2_ESM.png]

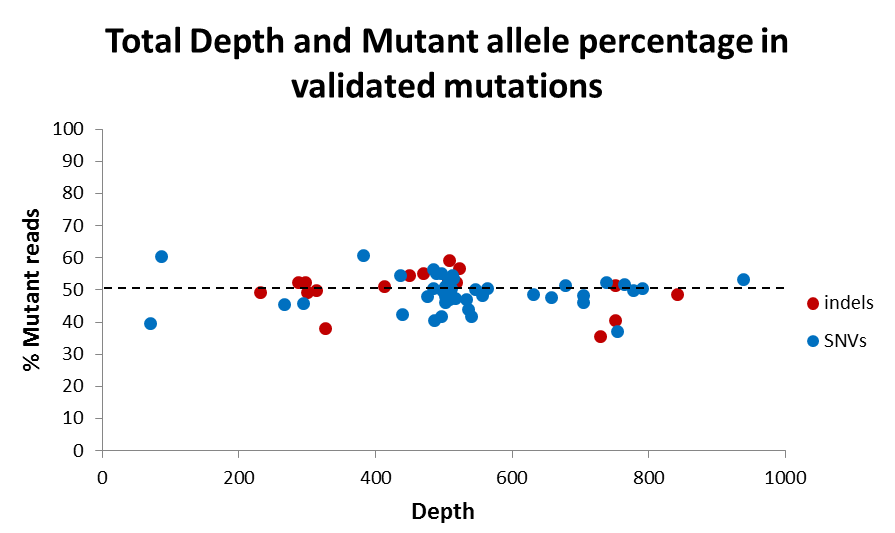

Supplement: Additional file 3: — Total depth and percentage of mutant reads in the validated heterozygote mutations (both previously knowns and unknowns). As expected, the percentage of mutant alleles approximated 50 % for both SNPs and indels. (PNG 17 kb) [file 12920_2016_184_MOESM3_ESM.png]
